# Coagulation factor VII and malignant progression of hepatocellular carcinoma

**DOI:** 10.1038/cddis.2015.395

**Published:** 2016-02-25

**Authors:** K-D Chen, K-T Huang, M-C Tsai, C-H Wu, I-Y Kuo, L-Y Chen, T-H Hu, C-L Chen, C-C Lin

**Affiliations:** 1 Institute for Translational Research in Biomedicine, Liver Transplantation Program and Division of General Surgery, Department of Surgery, Kaohsiung Chang Gung Memorial Hospital, Kaohsiung, Taiwan; 2 Division of Hepato-Gastroenterology, Department of Internal Medicine, Kaohsiung Chang Gung Memorial Hospital, Kaohsiung, Taiwan; 3 Graduate Institute of Clinical Medical Sciences, Chang Gung University, College of Medicine, Kaohsiung, Taiwan

In this invited News and Commentary article, we discuss our new paper ‘Factor VII promotes hepatocellular carcinoma progression through ERK–TSC signaling' in *Cell Death Discovery* (ref. 8).

Coagulation factor VII (FVII) is engaged in the initiation of the extrinsic coagulation pathway by binding to tissue factor (TF), and both factors have been considered as alternative therapeutic targets in hemostatic disorders and specific types of cancer.^1, 2^ It is universally accepted that FVII is manufactured by liver cells and circulates in the bloodstream, and only ~1% of total FVII circulates as an active enzyme (FVIIa), which is insufficient to initiate coagulation under physiological conditions.^3^ The presence of procoagulant TF increases the conversion of inactive FVII to the activated two-chain form, and this triggers the coagulation serine protease cascade when FVIIa forms a binary complex with the extracellular domain of TF. In addition to the central role in initiation of coagulation, recent studies have shown that ectopic synthesis of FVII by cancer cells promotes cell migration and metastasis.^4^ Others have further indicated that reduction of TF and FVIIa exerts an inhibitory effect on tumor growth in xenograft models of breast and colorectal cancer.^5, 6^ We have previously demonstrated that TF/FVII action triggers proteolytic cleavage and activation of protease-activated receptor 2 (PAR2), which in turn regulates autophagy mainly via mTOR signaling and impacts on cell survival of hepatocellular carcinoma (HCC) cells *in vitro* and *in vivo*.^7^ However, the role of TF and FVII in progression of HCC has remained elusive.

In our latest publication in *Cell Death Discovery*, despite a wide variation in the expression of TF, FVII and PAR2 in HCC tumors and non-tumor tissues, we observed a significant positive correlation between the expression of FVII and PAR2 in tumor specimens, and an association between FVII and the clinical staging.^8^ Furthermore, patients with high levels of FVII in HCC tissue had worse disease-free survival than those with low levels of FVII. Importantly, expression of FVII was exclusively associated with the presence of PAR2 through ERK–TSC signaling, but not downstream products of coagulation, such as thrombin and its signal transduction effector PAR1 (Figure 1). Therefore, the clinical observations suggest that FVII may have an important role in progression of HCC through a PAR2-dependent signaling pathway.

Our *in vitro* data further confirmed that FVII, but not soluble TF, activates ERK1/2 through PAR2. Moreover, the invasion- and migration-associated phenotypes could be effectively abolished by silencing FVII expression in HCC cells. We speculate that TF is constitutively expressed in HCC tissue, whereas the amount of FVIIa determines the proportion of TF that is engaged in the binary complex to regulate HCC progression. Our data from the mouse xenograft model showed that injection of FVIIa increased vascular density, but not the size and number of the tumors. These results were consistent with our clinical findings, in which the expression of FVII by HCC was associated with vascular invasion and capsulations of tumor but not the number and size. TF, nevertheless, could have an important role in tumor progression in many but not all cancers. Although our previous findings have shown that TF regulates survival of HCC cells via antagonizing autophagy through mTOR signaling, our current data suggest that TF may be, at least in our hands, less crucial in HCC progression.

It has been generally accepted that tumor-cell motility is necessary for cancer dissemination.^9^ The molecular basis for invading tumor cells to acquire the ability to colonize other organs has been long studied, but it still remains a largely unmet challenge in therapeutic control on metastatic dissemination.^10, 11^ In China and other East Asian countries, survival of HCC patients has greatly improved due to the advances in surgical techniques, such as orthotopic liver transplantation and perioperative nursing care. However, long-term survival after surgical resection remains low owing to risk of recurrence and metastasis.^12^ Thus, there is an urgent need to identify impaired metastatic suppressors for development of novel therapeutic strategies that supplement existing protocols.

In this study, we have shown that knockdown of FVII and PAR2 significantly reduced HCC invasion and migration. Inhibition of FVII–PAR2 signaling may thus represent an effective approach to targeted cancer therapy. Although there is potential risk of bleeding using specific FVIIa inhibitors,^5^ a recent phase I study indicated that PCI-27483, a selective inhibitor of FVIIa, which improves prothrombin time upto 2.5–3.0 folds in animal studies, is nevertheless well tolerated in advanced pancreatic cancer patients.^13^ Although the phase II trial is still ongoing, based on our current results, this particular agent may be a good candidate for therapeutic use in HCC. Furthermore, we showed that metastatic suppressors, NME/NM23 nucleoside diphosphate kinase 1 (NME1) and the basic helix–loop–helix family member e41 (BHLHE41), were highly induced in FVII and PAR2 knockdown HCC cells. Similarly, treatment with FVII and a PAR2 agonist significantly decreased expression of NME1 and BHLHE41. These results support the idea of targeting the FVII–PAR2 pathway for treatment of metastatic HCC. In summary, we have presented for the first time that increased FVII expression in tumor cells correlates with progression of HCC and acts as a poor prognostic factor after surgery, and demonstrated that FVII/PAR2 signaling through ERK1/2 is involved in HCC progression. Importantly, we have provided mechanistic insights showing that the FVII/PAR2 pathway not only affects mTOR for cell survival but also modulates migratory and metastatic properties in HCC.

## Figures and Tables

**Figure 1:**
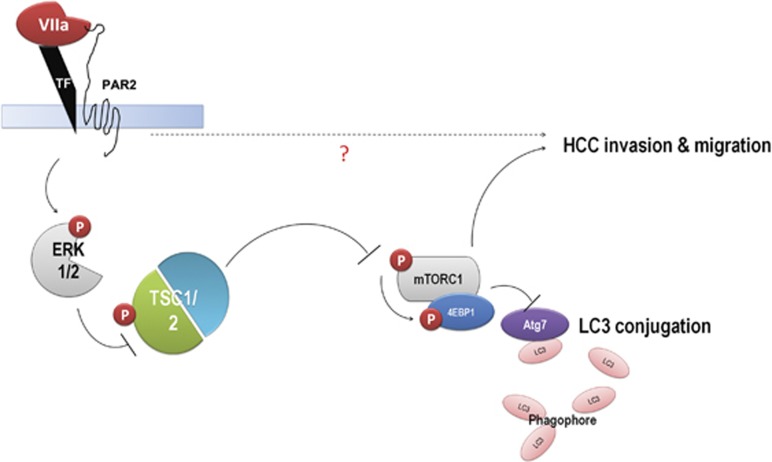
Scheme showing PAR2 transduces the FVII coagulation signal through the ERK–TSC pathway in the regulation of mTOR-dependent HCC progression
